# Social capital dimensions are differentially associated with COVID-19 vaccinations, masks, and physical distancing

**DOI:** 10.1371/journal.pone.0260818

**Published:** 2021-12-09

**Authors:** Ibtihal Ferwana, Lav R. Varshney

**Affiliations:** 1 Department of Computer Science, University of Illinois Urbana-Champaign, Urbana, IL, United States of America; 2 Department of Electrical and Computer Engineering, University of Illinois Urbana-Champaign, Urbana, IL, United States of America; 3 Coordinated Science Laboratory, University of Illinois Urbana-Champaign, Urbana, IL, United States of America; Konkuk University, REPUBLIC OF KOREA

## Abstract

**Background:**

Social capital has been associated with health outcomes in communities and can explain variations in different geographic localities. Social capital has also been associated with behaviors that promote better health and reduce the impacts of diseases. During the COVID-19 pandemic, social distancing, face masking, and vaccination have all been essential in controlling contagion. These behaviors have not been uniformly adopted by communities in the United States. Using different facets of social capital to explain the differences in public behaviors among communities during pandemics is lacking.

**Objective:**

This study examines the relationship among public health behavior—vaccination, face masking, and physical distancing—during COVID-19 pandemic and social capital indices in counties in the United States.

**Methods:**

We used publicly available vaccination data as of June 2021, face masking data in July 2020, and mobility data from mobile phones movements from the end of March 2020. Then, correlation analysis was conducted with county-level social capital index and its subindices (family unity, community health, institutional health, and collective efficacy) that were obtained from the Social Capital Project by the United States Senate.

**Results:**

We found the social capital index and its subindices differentially correlate with different public health behaviors. Vaccination is associated with institutional health: positively with fully vaccinated population and negatively with vaccination hesitancy. Also, wearing masks negatively associates with community health, whereases reduced mobility associates with better community health. Further, residential mobility positively associates with family unity. By comparing correlation coefficients, we find that social capital and its subindices have largest effect sizes on vaccination and residential mobility.

**Conclusion:**

Our results show that different facets of social capital are significantly associated with adoption of protective behaviors, e.g., social distancing, face masking, and vaccination. As such, our results suggest that differential facets of social capital imply a Swiss cheese model of pandemic control planning where, e.g., institutional health and community health, provide partially overlapping behavioral benefits.

## Introduction

Social capital has been developed as a concept to characterize the value of a community structure [1, 2]. To better reflect the nature of communities, social capital has further been defined as the quality of the relationship among community members, which is represented in trust and reciprocal aid that derive mutual benefits to all parties [3]. It has now become widely used to understand social determinants of public health [4, 5]. In particular, social capital in communities has been associated with health outcomes, such as mortality rate, obesity, and diabetes [6, 7] and can explain the variation in health status across different geographic areas [3]. For the United States, social capital has been operationalized and measured on county and state levels [8, 9].

Social capital is measured using several social elements that each reflect a different aspect of a community [8, 9]. These facets of social life, such as family unity and institutional trust, can further explain specific social outcomes or behaviors. For example, social capital stemming from family support has been associated with better mental health [10], better mechanisms for coping with stress [11], and lower suicide rates [12]. Social capital stemming from civic participation, such as taking part in religious or volunteer groups, promoted better sense of responsibility, and in turn created healthier neighborhoods and higher levels of life satisfaction [13]. It may not be surprising that social capital may influence public behaviors related to health, however, how social capital does so and what it implies about safety is an open question, which we are interested in further examining in this study.

### Social capital and COVID-19

After the declaration of COVID-19 as a pandemic [14], social distancing and wearing masks were recommended as non-pharmaceutical interventions to contain the spread. Even though recommendations were widely announced and justified, not all communities abided uniformly to the new recommendations. Some communities increased an individual sense of responsibility to take actions, e.g., social distancing, to protect self and others [15], whereas other communities found it difficult to isolate and eliminate social gatherings [16, 17]. Thus, growth of COVID-19 differed among communities and has been associated with social capital and some of its dimensions. The number of COVID-19 confirmed cases decreased with better community health [18], whereases mortality rate increased with lower social capital levels [19], and lack of institutional trust and civic engagement [17]. Collective adherence to protective behaviors during a pandemic might mitigate the critical consequences of its spread.

### Physical distancing

Since COVID-19 is highly infectious and transmits easily with face-to-face interaction, social distancing proved to be an effective mitigation strategy to contain COVID-19 spread [20]. Cases decrease by 48% and fatalities by 60% three weeks after states implemented lockdown orders [21]. Physical distancing strategies took a variety of forms, from limiting people’s gatherings to fully restricting movements by lockdown orders. In the United States, there were distinctive patterns in mobility reduction among different sociodemographic groups, where some communities voluntarily stayed at home and limited their movements even more after lockdown orders [22].

### Wearing masks

Face masking also has been an effective non-pharmaceutical intervention which lowers the risks of testing positive for COVID-19 infection by 70% [23, 24]. In April 2020, with the absence of vaccines, the Centers for Disease Control and Prevention (CDC) recommended the use of cloth masks in public [25], especially after finding that infectious microbes of COVID-19 can be transmitted from persons without symptoms [26, 27]. All sociodemographic groups adopted mask wearing but there were larger increases in specific geographic areas such as the Midwest, and 76% of the population wears masks when leaving their homes [28].

### Vaccination

For pandemic extinction, it is believed that 70% to 80% of the population must be vaccinated [29, 30] and the threshold decreases with following protective health behaviors, such as face masking and social distancing [31]. At the initial stage of vaccination development in 2020, half of the U.S. population did not intend to take the vaccine because of health concerns and low confidence in the vaccine [32, 33]. Also, media misinformation about vaccination strongly lowered people’s intention to be vaccinated, and some sociodemographic groups were impacted differently [34]. However, the hesitancy against vaccination started to decline by May 2021 among all demographic groups [35]. As hesitancy declined, about 51% of the U.S. population are fully vaccinated as of August 2021 [36].

Social responses toward pandemics are critical in containing the spread and mitigating its exacerbated effects. The way communities are structured impacts individuals’ ability to adopt new behaviors, and hence, follow public health recommendations [37]. This study aims to explore the association, if any, between different social capital facets and public health behaviors: social distancing, wearing masks and vaccination, during the COVID-19 pandemic at the county-level in the United States.

## Data and methods

We estimate the effects of social capital on public health behaviors related to COVID-19 pandemic. Table 1 summarizes the variables we used in the analysis with descriptive statistics at the county-level.

**Table 1:** List of variables and descriptives.

Variable	Notes	Mean	SD	Minimum	Maximum
**Social Capital**	Standardized	0.00	1.00	-4.32	2.97
**Family Unity**	Standardized	0.00	1.00	-4.93	2.66
**Community Health**	Standardized	0.00	1.00	-1.67	7.07
**Institutional Health**	Standardized	0.00	1.00	-4.66	2.99
**Collective Efficacy**	Standardized	0.00	1.00	-8.42	1.22
**Fully Vaccination**	Cummulative percentage of population as of June 2021	28.96	14.39	0.00	99.90
**Vaccine Hesitancy Rate**	Cummulative percentage of population as of June 2021	13.18	4.21	3.81	25.61
**Always Wearing Masks**	Percentage of population on July 2020	50.81	15.22	11.50	88.90
**Never Wearing Masks**	Percentage of population on July 2020	7.99	5.85	0.00	43.20
**Mobility Index**	Week of 23 March 2020	3.18	0.53	0.65	4.16
**Retail and Recreation (percent change from baseline)**	Week of 23 March 2020	-35.84	13.43	-91.29	112.80
**Residential (percent change from baseline)**	Week of 23 March 2020	15.68	3.83	7.33	31.43

We obtained county-level social capital indices from the Social Capital Project [9], which comprise four subindices: *family unity* which considers the structure of families in terms of marriage and children; *community health* which considers participation in civic life such as involvement in volunteering and religious groups; *institutional health* which considers confidence in media/corporations/schools, and participation in institutions such as elections and census; and *collective efficacy* which is the converse of social disorganization, operationalized via violent crime rates.

To assess public health behavior, we considered vaccination rate, vaccination hesitancy, mask usage, and changes in mobility patterns at relevant times during COVID-19. We used county-level data considering the fully vaccinated population [38], and estimated vaccine hesitancy [36] as of June 2021. County-level mask usage data is based on a survey of 250,000 people conducted between July 2-14, 2020 [39]: we consider extreme responses of “never” and “always”. County-level mobility index is computed by Cuebiq firm [40] based on changes in mobile phone movement. Changes in retail and recreation, and residential mobility are obtained from Google Community Mobility Reports [41]. We consider the March 23, 2020 week for mobility data before wide mandates of lockdowns were issued.

The bivariate relationships between each social capital index and public health behavior are assessed using standardized linear regression. Statistical analysis along with P-values and 95% confidence intervals are reported. All analyses have been done using statsmodels package in Python [42].

Aggregated mobility data is provided by Cuebiq, a location intelligence and measurement platform. Through its Data for Good program, Cuebiq provides access to aggregated mobility data for academic research and humanitarian initiatives. This first-party data is collected from anonymized users who have opted-in to provide access to their location data anonymously, through a GDPR-compliant framework. It is then aggregated to the census-block group level to provide insights on changes in human mobility over time. All other datasets used in this study are publicly available and may be used for academic research, as per their terms and conditions.

## Results

**Fig 1:**
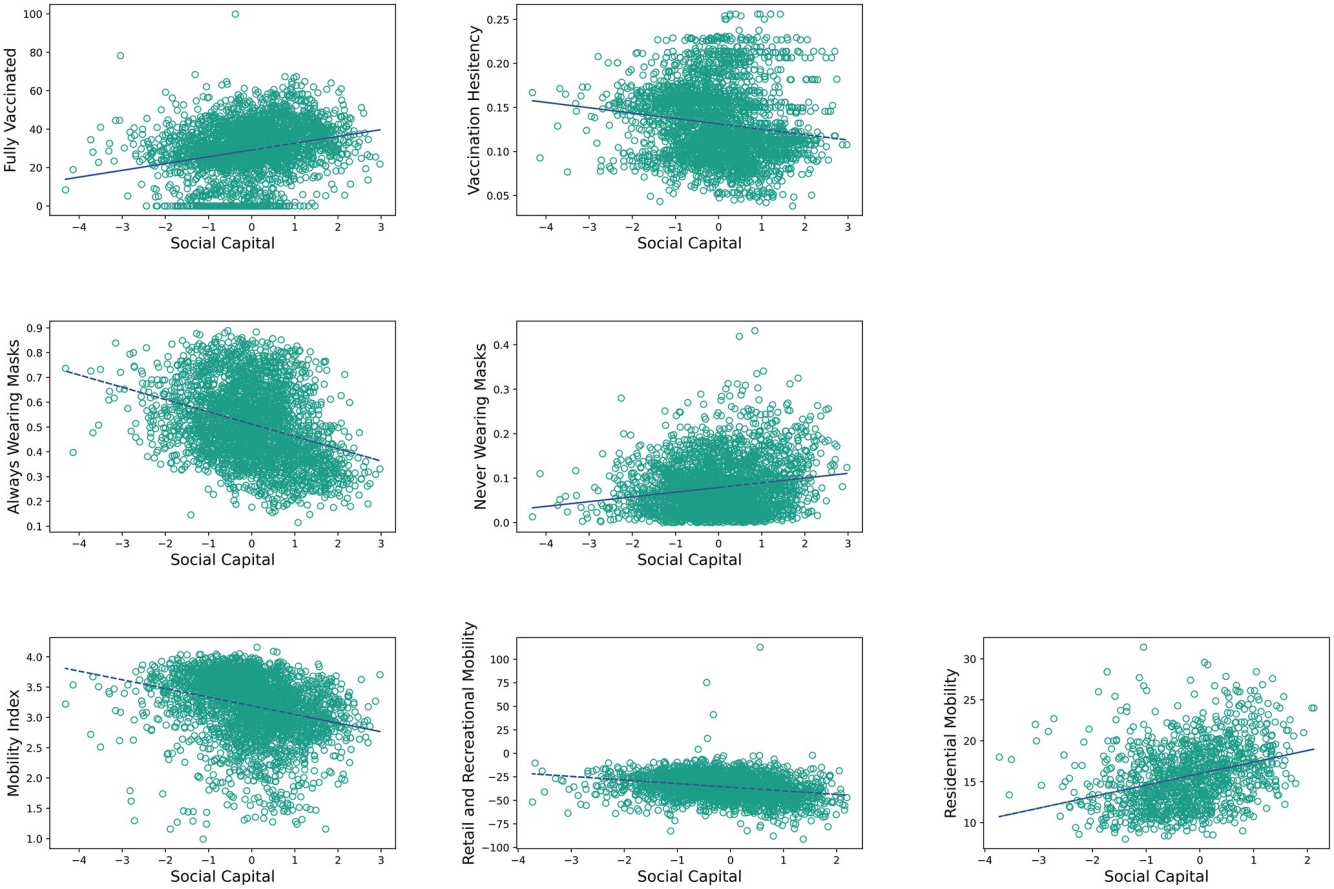
Bivariate correlation for each public health behavior and the main social capital index. (Effect sizes, confidence intervals, p-values and R2 are reported in Table 2).

By looking at the aggregated social capital index, we see that it significantly associates with each public behavior, positively with vaccination, negatively with COVID-19 vaccination hesitancy, negatively with masking, and positively with reduced mobility, as illustrated in Fig 1 with further statistical details in Table 2. This supports previous studies findings on the relationship between social capital and health factors and behaviors [4, 7, 18]. To further understand the relationship between different dimensions of social capital, e.g., trust, family unity, community engagement and public behaviors we further examine social capital subindices.

**Table 2:** Bivariate correlation estimation table. Each line represents a regression analysis between a public health behavior and social capital index.

Public Health Behavior	Social Capital Index	Coefficient (95% CI)	p-value	*R* ^2^	*N*
**Fully Vaccination**	Social Capital	0.248 (0.21 to 0.28)	2.6 × 10^−43^	0.0617	2991
	Family Unity	0.113 (0.08 to 0.15)	5.18 × 10^−10^	0.0127	3020
	Community Health	0.146 (0.11 to 0.18)	1.95 × 10^−16^	0.0213	3138
	Institutional Health	0.33 (0.3 to 0.36)	6.99 × 10^−80^	0.1088	3111
	Collective Efficacy	0.065 (0.03 to 0.1)	0	0.0043	3022
**Vaccine Hesitancy Rate**	Social Capital	-0.148 (-0.18 to -0.11)	4.59 × 10^−16^	0.0218	2992
	Family Unity	-0.128 (-0.16 to -0.09)	1.95 × 10^−12^	0.0163	3021
	Community Health	0.003 (-0.03 to 0.04)	0.85	0	3139
	Institutional Health	-0.261 (-0.29 to -0.23)	1.54 × 10^−49^	0.068	3112
	Collective Efficacy	0.001 (-0.03 to 0.04)	0.95	0	3023
**Always Wearing Masks**	Social Capital	-0.329 (-0.36 to -0.3)	1.56 × 10^−76^	0.1083	2992
	Family Unity	-0.19 (-0.23 to -0.16)	5.68 × 10^−26^	0.0361	3021
	Community Health	-0.37 (-0.4 to -0.34)	1.27 × 10^−102^	0.1371	3139
	Institutional Health	-0.19 (-0.22 to -0.15)	1.49 × 10^−26^	0.0359	3112
	Collective Efficacy	-0.201 (-0.24 to -0.17)	7.28 × 10^−29^	0.0403	3023
**Never Wearing Masks**	Social Capital	0.184 (0.15 to 0.22)	4.15 × 10^−24^	0.0337	2992
	Family Unity	0.094 (0.06 to 0.13)	2.45 × 10^−7^	0.0088	3021
	Community Health	0.267 (0.23 to 0.3)	1.88 × 10^−52^	0.0714	3139
	Institutional Health	0.097 (0.06 to 0.13)	6.58 × 10^−8^	0.0093	3112
	Collective Efficacy	0.105 (0.07 to 0.14)	7.02 × 10^−9^	0.011	3023
**Mobility Index**	Social Capital	-0.276 (-0.31 to -0.24)	2.56 × 10^−53^	0.076	2992
	Family Unity	-0.214 (-0.25 to -0.18)	1.39 × 10^−32^	0.0457	3021
	Community Health	-0.267 (-0.3 to -0.23)	1.65 × 10^−52^	0.0715	3139
	Institutional Health	-0.233 (-0.27 to -0.2)	1.26 × 10^−39^	0.0543	3112
	Collective Efficacy	-0.09 (-0.13 to -0.05)	7.18 × 10^−7^	0.0081	3023
**Retail and Recreation Mobility**	Social Capital	-0.267 (-0.3 to -0.23)	4.59 × 10^−42^	0.0712	2503
	Family Unity	-0.204 (-0.24 to -0.17)	5.19 × 10^−25^	0.0416	2516
	Community Health	-0.231 (-0.27 to -0.19)	2.57 × 10^−32^	0.0535	2548
	Institutional Health	-0.264 (-0.3 to -0.23)	1.12 × 10^−41^	0.0697	2535
	Collective Efficacy	-0.075 (-0.11 to -0.04)	0	0.0056	2505
**Residential Mobility**	Social Capital	0.322 (0.27 to 0.37)	3.51 × 10^−33^	0.104	1314
	Family Unity	0.36 (0.31 to 0.41)	1.36 × 10^−41^	0.1298	1315
	Community Health	0.021 (-0.03 to 0.07)	0.45	0.0004	1317
	Institutional Health	0.322 (0.27 to 0.37)	4.41 × 10^−33^	0.1035	1316
	Collective Efficacy	0.158 (0.1 to 0.21)	9.11 × 10^−9^	0.025	1309

**Fig 2:**
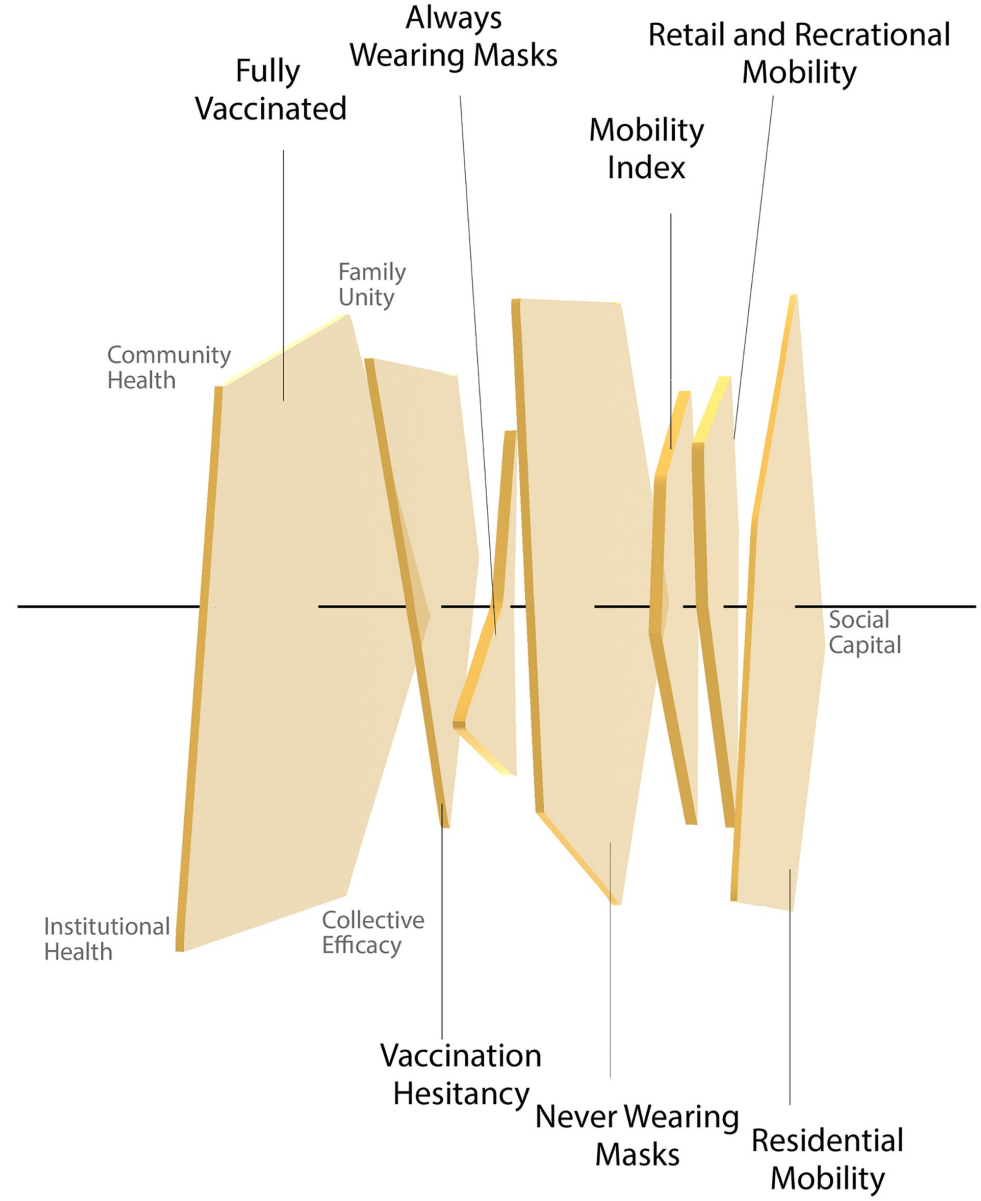
A Swiss cheese model for social capital, with pentagons representing the five social capital indices’ impact on social behaviors.

**Fig 3:**
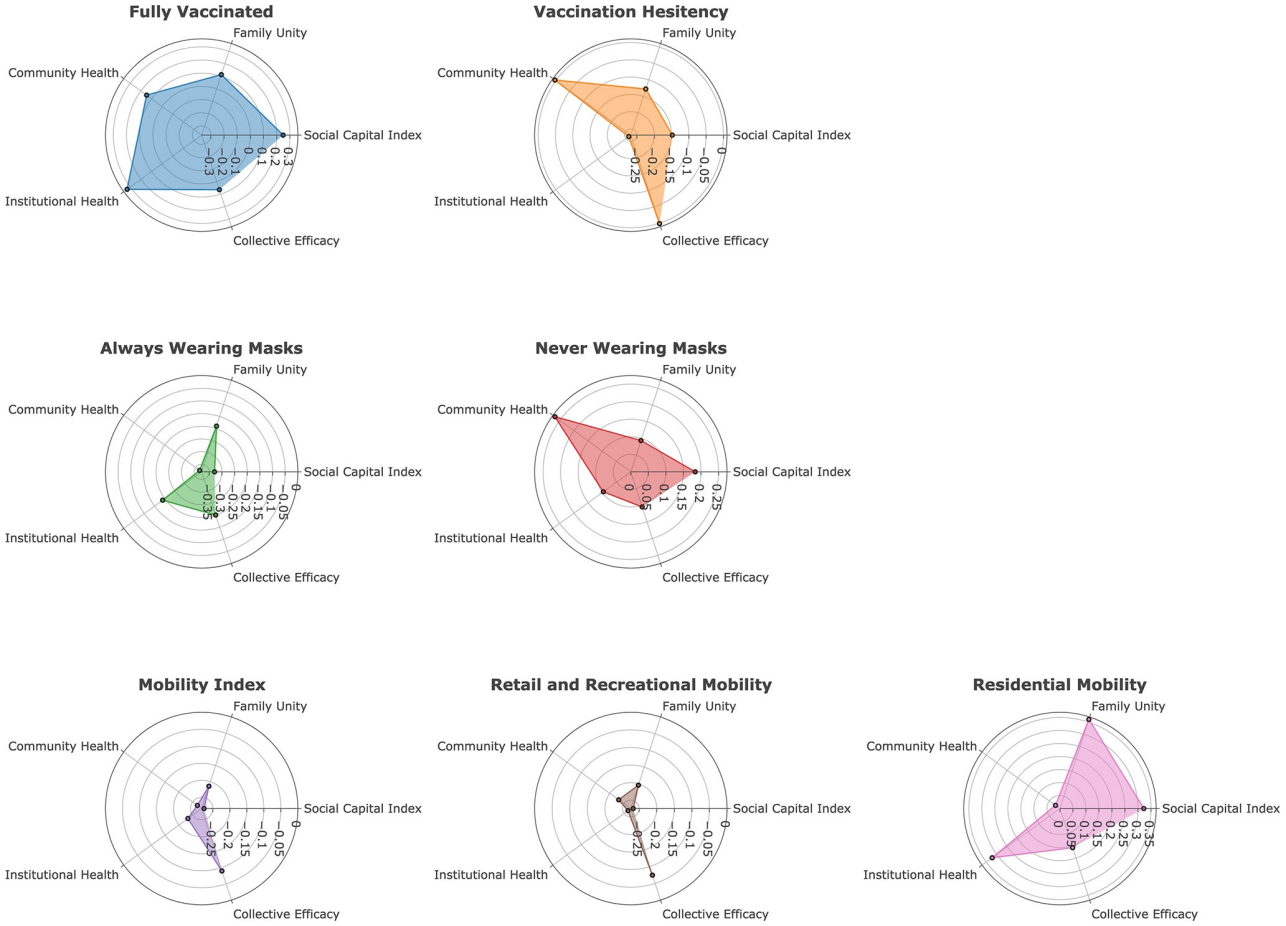
Bivariate correlation for each public behavior against social capital indices represented in radar charts. (Effect sizes, confidence intervals, p-values and R^2^ are reported in Table 2).

Social capital subindices associate differently with COVID-19 vaccination, masking, and mobility change behaviors, illustrated in the Swiss cheese model [43, 44] that we extend to social capital (Fig 2). Each public behavior affected by different social capital facets resembles a defensive layer against the spread of COVID-19. The Swiss cheese model is created from the radar charts shown in Fig 3.

Fig 3 specifies the differences in correlation sizes for each public health behavior against social capital and its four subindices. Correlation coefficients of full vaccination are the largest, and of residential mobility are the second largest, whereas mobility and mask usage have smaller effect sizes. Family unity has similar effect sizes for all public health behaviors except largest for the mobility index. Community health has greatest effect size for masking, less for vaccination, and least for the mobility indices. Institutional health has greatest effect size for vaccination and change in residential mobility but smallest in masking. Collective efficacy has smallest effect size for mobility and largest effect size for mask wearing.

**Fig 4:**
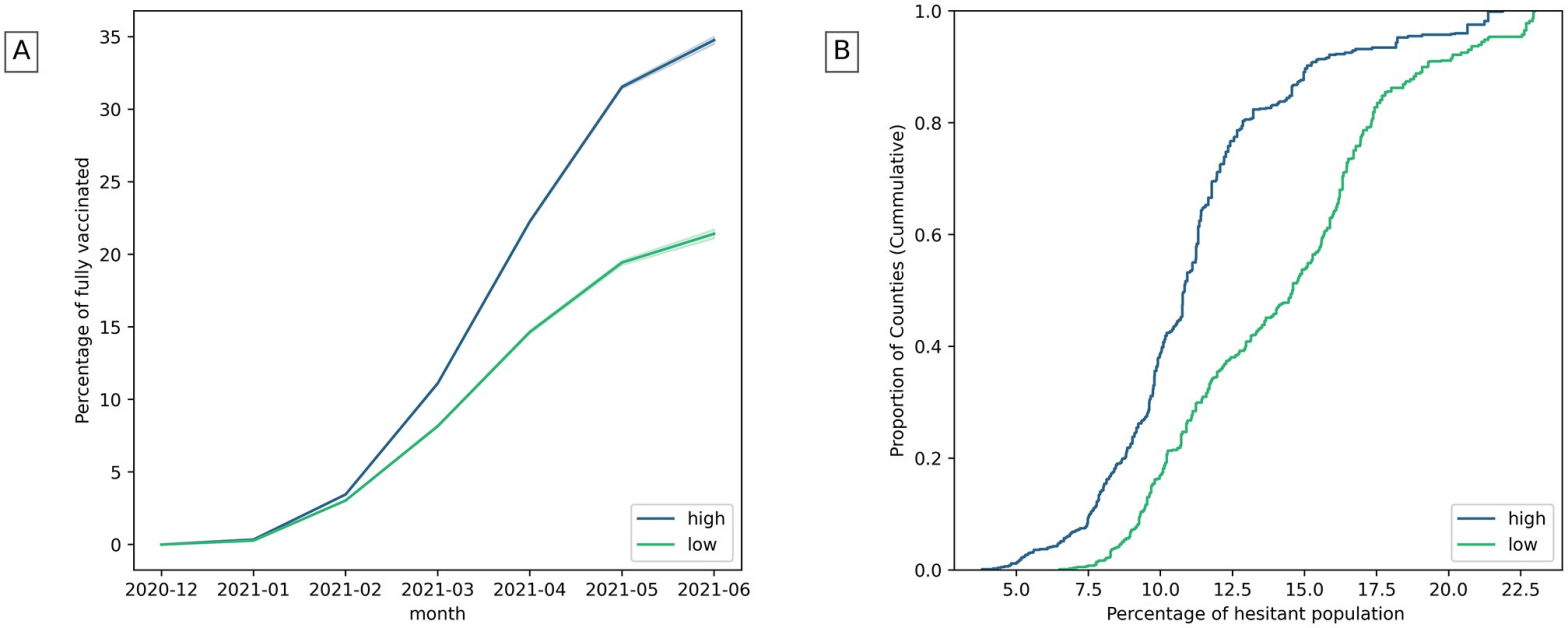
A) Fully vaccinated population over time in counties with high and low institutional health. B) Estimated hesitant population in counties with high and low institutional health. (The highest and lowest 25% of counties are considered).

Vaccination mostly associates significantly with institutional health, positively with fully vaccinated population, but negatively with increased hesitant population (Table 2). Fig 4A shows that over time, counties with high institutional health have an increasing rate of vaccination unlike counties with lower institutional health. Further, Fig 4B shows that hesitant population is less in counties with higher institutional health.

**Fig 5:**
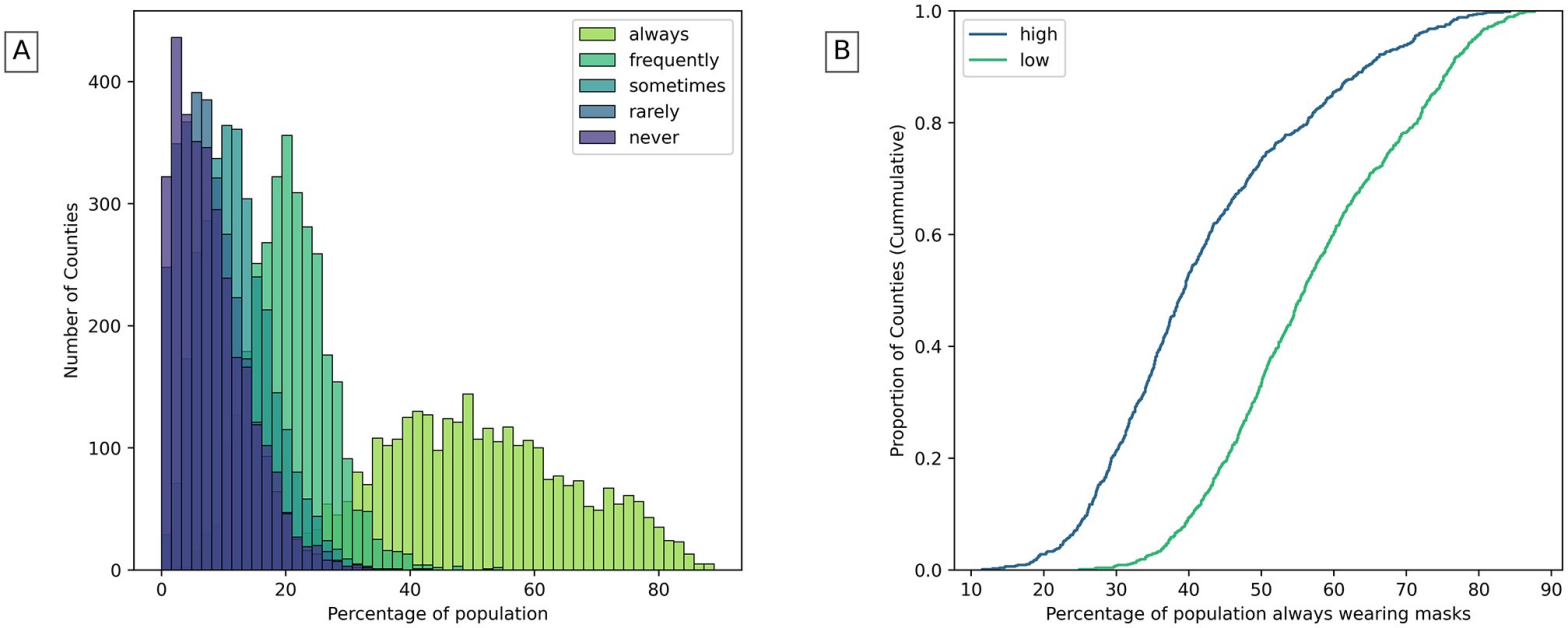
A) Population that wears masks in counties. B) Population that always wears masks in counties with high and low community health (The highest and lowest 25% of counties are considered).

Most counties have individuals who constantly wear masks, while fewer counties have people who rarely wear masks (Fig 5A). Wearing masks mostly associate with community health, positively with reduced masks usage and negatively with widely mask usage (Table 2). Fig 5B shows that counties with higher community health have less people who always wear masks.

**Fig 6:**
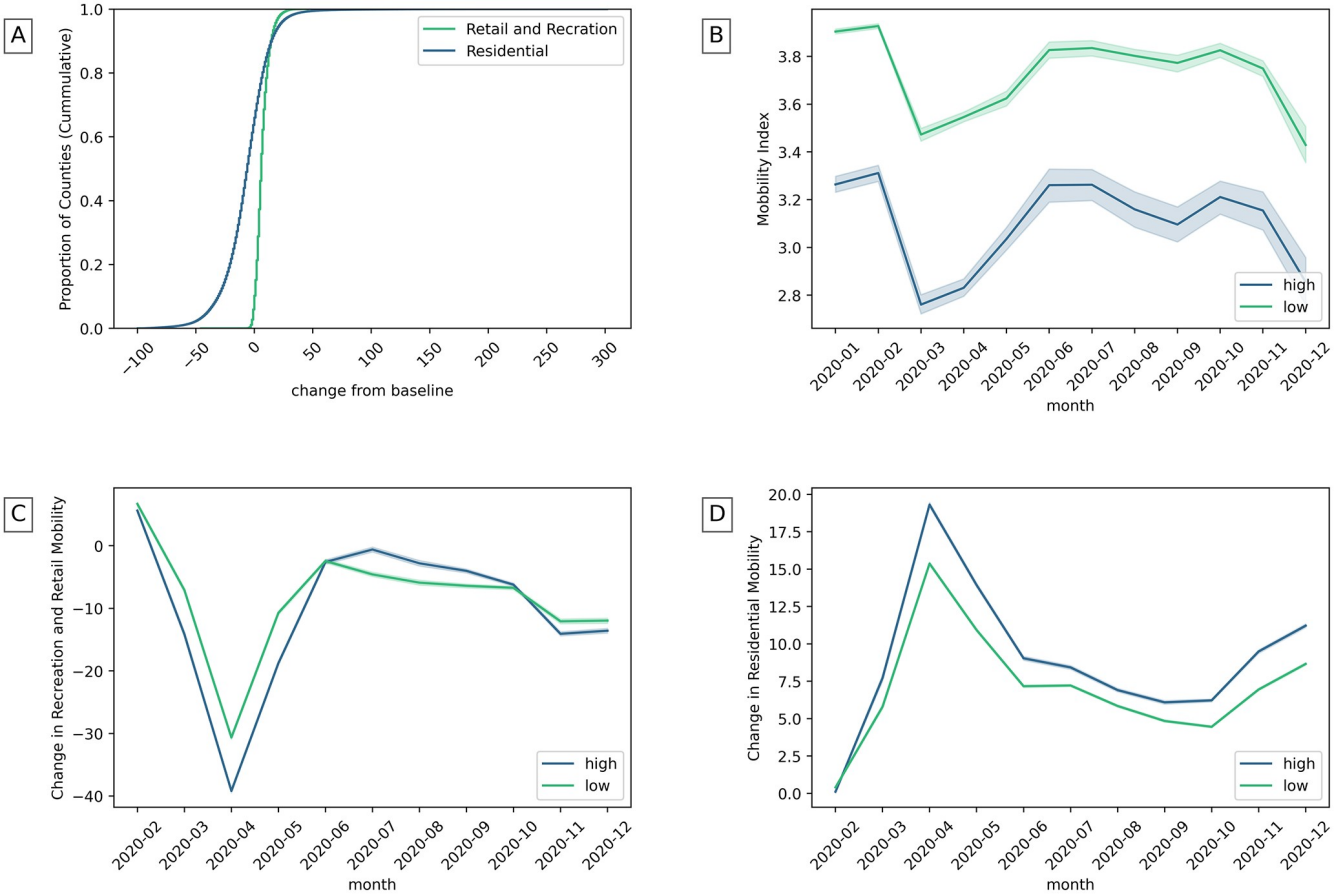
A) The change of recreation and residential mobility in counties. B) Mobility index in counties with high and low community health. C) Recreation and retail mobility in counties with high and low institutional health. D) Residential mobility with high and low family unity. (The highest and lowest 25% of counties are considered).

In general, people reduced their visits in recreational areas more than in residential areas (Fig 6A). Reduced mobility associates mostly with higher overall social capital and secondly with community health (Table 2). Fig 6B shows that counties with better community health tend to move less. Reduced recreational mobility, as well, associate mostly with higher overall social capital (Table 2) and secondly with better institutional health (Fig 6C). While increased residential mobility associates mostly with higher family unity (Table 2) as seen in Fig 6D.

## Discussion

During pandemics, social capital and its dimensions play a role in differentiating public responses towards health policies and interventions, and in turn health outcomes diverge. Trusting institutions reduced anxiety during SARS pandemic [45], and predicted vaccination acceptance during H1N1 pandemic [46, 47]. Also, people’s intentions to wearing masks and washing hands increased with better social capital [7].

Similarly, during the COVID-19 pandemic, behavioral responses such as vaccination, masking, and physical distancing have differed among United States counties. Here we have shown that facets of social capital are associated with behavioral responses to the COVID-19 pandemic in different ways. Our findings show that trusting institutions may promote vaccination and reduce vaccination hesitancy. Also, communities with more engagement in civic life tend to reduce their mobility. This supports the findings of [48], where communities with higher civic life engagement increased the sense of responsibility in individuals to lower their gatherings. However, higher community health may motivate people to relax their face masking, and this can be explained with overall reduced mobility and less face-to-face interactions. People stay home with better family unity whereas recreational visits decrease with better social capital and institutional health.

Our results suggest that social capital and its subindices are essential in explaining differences in public behaviors during health crises which may help determine policies in local communities. Further, our results show that differential facets of social capital imply a Swiss cheese model of pandemic control planning where multiple layers of public behaviors differently affected by social capital can act against contagion spread. There might be some barriers, such as community structure, misinformation from media, and medical concerns, for an individual to adopt new behaviors in pandemics. Therefore, more effort might be needed to help individuals to adhere to new protective behaviors especially in communities that generally have lower social capital.

## References

[1] Coleman JS. Social Capital in the Creation of Human Capital. *American Journal of Sociology*. 1988; 94: S95–S120. doi: 10.1086/228943

[2] Putnam RD. Bowling Alone: America’s Declining Social Capital. *Journal of Democracy*. 1995; 65–78. doi: 10.1353/jod.1995.0002

[3] Kawachi I. Social Capital and Community Effects on Population and Individual Health. *Annals of the New York Academy of Sciences*. 1999; 896: 120–130. doi: 10.1111/j.1749-6632.1999.tb08110.x 10681893

[4] Kawachi I, Subramanian SV, Kim D. Social capital and health. In Social capital and health 2008 (pp. 1–26). Springer, New York, NY.

[5] Lynch J, Due P, Muntaner C, et al. Social capital—Is it a good investment strategy for public health? *Journal of Epidemiology Community Health* 2000; 54: 404–408. doi: 10.1136/jech.54.6.404 10818113 PMC1731686

[6] Ehsana A, Klaas HS, Bastianen A, et al. Social capital and health: A systematic review of systematic reviews. *SSM—Pop H* 2019; 8: 100425. 10.1016/j.ssmph.2019.100425 PMC6580321 31431915

[7] Chuang YC, Huang YL, Tseng KC, Yen CH, Yang LH. Social capital and health-protective behavior intentions in an influenza pandemic. PLoS one. 2015 Apr 15;10(4):e0122970. doi: 10.1371/journal.pone.0122970 25874625 PMC4398366

[8] Rupasingha A, Goetz SJ, Freshwater D. The production of social capital in US counties. *The Journal of Socio-Economics* 2006; 35: 83–101. doi: 10.1016/j.socec.2005.11.001

[9] Lee M. The geography of social capital in America. *U.S. Congress Joint Economic Committee* 2018; 1–66.

[10] McKenzie K, Whitley R, Weich S. Social capital and mental health. *The British Journal of Psychiatry*. 2002 Oct;181(4):280–3. doi: 10.1192/bjp.181.4.280 12356653

[11] Berkman L, Syme L. Social networks, host resistance, and mortality: a nine-year follow-up study of Alameda County residents. *American Journal of Epidemiology*. 1979; 109: 186–204. doi: 10.1093/oxfordjournals.aje.a112674 425958

[12] Helliwell JF. Well-being and social capital: Does suicide pose a puzzle?. *Social Indicators Research*. 2007 May;81(3):455–96. doi: 10.1007/s11205-006-0022-y

[13] Elgar FJ, Davis CG, Wohl MJ, et al. Social capital, health and life satisfaction in 50 countries. *Health Place*. 2011; 17: 1044–1053. doi: 10.1016/j.healthplace.2011.06.010 21784694

[14] Cucinotta D, Vanelli M. WHO Declares COVID-19 a Pandemic. *Acta Biomed*. 2020; 91: 157–160. 32191675 10.23750/abm.v91i1.9397 PMC7569573

[15] Durante R, Guiso L, Gulino G. Asocial capital: Civic culture and social distancing during COVID-19. *Journal of Public Economics*. 2021; 194: 104342. doi: 10.1016/j.jpubeco.2020.104342 PMC9186120 35702335

[16] Ding W, Levine R, Lin C, Xie W. Social distancing and social capital: Why US counties respond differently to COVID-19. National Bureau of Economic Research; 2020 Jun 22.

[17] Elgar FJ, Stefaniak A, Wohl MJA. The trouble with trust: Time-series analysis of social capital, income inequality, and COVID-19 deaths in 84 countries. *Social Science and Medicine*. 2020; 263: 113365. doi: 10.1016/j.socscimed.2020.113365 32981770 PMC7492158

[18] Varshney LR, Socher R. COVID-19 Growth Rate Decreases with Social Capital. medRxiv April 2020. 10.1101/2020.04.23.20077321.

[19] Borgonovi F, Andrieu E, Subramanian SV. The evolution of the association between community level social capital and COVID-19 deaths and hospitalizations in the United States. *Social Science and Medicine* 2021; 278: 113948. doi: 10.1016/j.socscimed.2021.113948 33930677 PMC8055504

[20] Friedson AI, McNichols D, Sabia JJ, Dave D. Did California’s shelter-in-place order work? Early coronavirus-related public health effects. *National Bureau of Economic Research*; 2020 Apr 20.

[21] Fowler JH, Hill SJ, Levin R, Obradovich N. Stay-at-home orders associate with subsequent decreases in COVID-19 cases and fatalities in the United States. *PLoS ONE*. 2021 Jun 10;16(6):e0248849. doi: 10.1371/journal.pone.0248849 34111123 PMC8191916

[22] Lee M, Zhao J, Sun Q, Pan Y, Zhou W, Xiong C, et al. Human mobility trends during the early stage of the COVID-19 pandemic in the United States. *PLoS One*. 2020 Nov 9;15(11):e0241468. doi: 10.1371/journal.pone.0241468 33166301 PMC7652287

[23] Payne DC. SARS-CoV-2 Infections and Serologic Responses from a Sample of U.S. Navy Service Members—USS Theodore Roosevelt, April 2020. *MMWR Morbidity and Mortality Weekly Report* 2020; 69: 714–721. doi: 10.15585/mmwr.mm6923e4 32525850 PMC7315794

[24] Ueki H, Furusawa Y, Iwatsuki-Horimoto K, Imai M, Kabata H, Nishimura H, et al. Effectiveness of face masks in preventing airborne transmission of SARS-CoV-2. MSphere. 2020 Oct 28;5(5):e00637–20. doi: 10.1128/mSphere.00637-20 33087517 PMC7580955

[25] CDC Guidelines. COVID-19: Considerations for Wearing Masks | CDC, https://www.cdc.gov/coronavirus/2019-ncov/prevent-getting-sick/cloth-face-cover-guidance.html (2020, accessed August 9, 2021).

[26] Kimball A, Hatfield K, Arons M, et al. Asymptomatic and Presymptomatic SARS-CoV-2 Infections in Residents of a Long-Term Care Skilled Nursing Facility—King County, Washington, March 2020. *MMWR Morbidity and Mortality Weekly Report* 2020; 69: 377–381. doi: 10.15585/mmwr.mm6913e1 32240128 PMC7119514

[27] Bai Y, Yao L, Wei T, et al. Presumed Asymptomatic Carrier Transmission of COVID-19. *Journal of American Medical Association* 2020; 323: 1406–1407. doi: 10.1001/jama.2020.2565 32083643 PMC7042844

[28] Fisher KA. Factors Associated with Cloth Face Covering Use Among Adults During the COVID-19 Pandemic—United States, April and May 2020. *MMWR Morbidity and Mortality Weekly Report* 2020; 69: 933–937. doi: 10.15585/mmwr.mm6928e3 32673303

[29] Robinson E, Jones A, Lesser I, et al. International estimates of intended uptake and refusal of COVID-19 vaccines: A rapid systematic review and meta-analysis of large nationally representative samples. *Vaccine* 2021; 39: 2024–2034. 33722411 10.1016/j.vaccine.2021.02.005 PMC7867398

[30] Bartsch SM, O’Shea KJ, Ferguson MC, et al. Vaccine Efficacy Needed for a COVID-19 Coronavirus Vaccine to Prevent or Stop an Epidemic as the Sole Intervention. *American Journal of Preventive Medicine*. 2020; 59: 493–503. doi: 10.1016/j.amepre.2020.06.011 32778354 PMC7361120

[31] Iboi EA, Ngonghala CN, Gumel AB. Will an imperfect vaccine curtail the COVID-19 pandemic in the U.S.? *Infectious Disease Modelling*. 2020; 5: 510–524. doi: 10.1016/j.idm.2020.07.006 32835142 PMC7409819

[32] Szilagyi PG, Thomas K, Shah MD, et al. National Trends in the US Public’s Likelihood of Getting a COVID-19 Vaccine—April 1 to December 8, 2020. *JAMA* 2021; 325: 396–398. doi: 10.1001/jama.2020.26419 PMC7772743 33372943

[33] Soares P, Rocha JV, Moniz M, Gama A, Laires PA, Pedro AR, et al. Factors associated with COVID-19 vaccine hesitancy. Vaccines. 2021 Mar;9(3):300. doi: 10.3390/vaccines9030300 33810131 PMC8004673

[34] Loomba S, de Figueiredo A, Piatek SJ, et al. Measuring the impact of COVID-19 vaccine misinformation on vaccination intent in the UK and USA. *Nature Human Behaviour* 2021 5:3 2021; 5: 337–348. doi: 10.1038/s41562-021-01056-1 33547453

[35] Daly M, Jones A, Robinson E. Public Trust and Willingness to Vaccinate Against COVID-19 in the US From October 14, 2020, to March 29, 2021. *JAMA* 2021; 325: 2397–2399. doi: 10.1001/jama.2021.8246 34028495 PMC8145162

[36] Health and Human Services. Vaccine Hesitancy for COVID-19: County and local estimates | Data | Centers for Disease Control and Prevention, https://data.cdc.gov/Vaccinations/Vaccine-Hesitancy-for-COVID-19-County-and-local-es/q9mh-h2tw (2021, accessed July 13, 2021).

[37] van Bavel JJ, Baicker K, Boggio PS, et al. Using social and behavioural science to support COVID-19 pandemic response. *Nature Human Behaviour* 2020; 4: 460–471. doi: 10.1038/s41562-020-0884-z 32355299

[38] Immunization and Information Systems. COVID-19 Vaccinations in the United States, Jurisdiction | Data | Centers for Disease Control and Prevention, https://data.cdc.gov/Vaccinations/COVID-19-Vaccinations-in-the-United-States-Jurisdi/unsk-b7fc (2021, accessed July 13, 2021).

[39] The New York Times, Dynata. Mask-Wearing Survey Data. *New York Times*, https://github.com/nytimes/covid-19-data/blob/master/mask-use/README.md (2020, accessed July 13, 2021).

[40] Cuebiq. Mobility Data, https://www.cuebiq.com/

[41] Google. COVID-19 Community Mobility Reports, https://www.google.com/covid19/mobility/ (2020, accessed August 10, 2021).

[42] Seabold S, Perktold J. statsmodels: Econometric and statistical modeling with python. *9^th^ Python in Science Conference*. 2010.

[43] Reason J. Human error: models and management. Bmj. 2000 Mar 18;320(7237):768–70. doi: 10.1136/bmj.320.7237.768 10720363 PMC1117770

[44] Larouzee J, le Coze JC. Good and bad reasons: The Swiss cheese model and its critics. *Safety Science* 2020; 126: 104660. doi: 10.1016/j.ssci.2020.104660

[45] Cheung C-K, Tse JW. Institutional trust as a determinant of anxiety during the SARS crisis in Hong Kong. *Social Work in Public Health* 2008; 23: 41–54. doi: 10.1080/19371910802053224 19301537

[46] Freimuth VS, Musa D, Hilyard K, Quinn SC, Kim K. Trust during the early stages of the 2009 H1N1 pandemic. Journal of health communication. 2014 Mar 1;19(3):321–39. doi: 10.1080/10810730.2013.811323 24117390 PMC3943629

[47] Björn R. Social capital and immunization against the 2009 A(H1N1) pandemic in the American States. *Public health* 2014; 128: 709–715. doi: 10.1016/j.puhe.2014.05.015 25132391

[48] Borgonovi F, Andrieu E. Bowling together by bowling alone: Social capital and Covid-19. *Social science and medicine*. 2020 Nov 1;265:113501. doi: 10.1016/j.socscimed.2020.113501 33203551

